# Severe Asthma Exacerbations in the Pediatric Intensive Care Unit: Clinical Profile, Management, and Outcomes—Retrospective Study

**DOI:** 10.3390/children13050710

**Published:** 2026-05-21

**Authors:** Amal H. Aljohani, Hamdi Ahmed Alsufiani, Abeer Musaibieh AlSaadi, Nora Abdulrahman Alem, Mamoun AliAbusunoon, Amnah Ibrahim Madkhali

**Affiliations:** 1 Division of Allergy and Clinical Immunology, Pediatric Department, College of Medicine, Taibah University, Madinah 42353, Saudi Arabia; 2 Division of Pulmonary Medicine, Pediatric Department, King Salman Medical City, Madinah 42319, Saudi Arabiaabmualsaadi@moh.gov.sa (A.M.A.); naalem@moh.gov.sa (N.A.A.); 3 Clinical Science Department, College of Medicine, Al-Rayan National Colleges, Madinah 42541, Saudi Arabia; 4 Pediatric Department, King Salman Medical City, Madinah 42319, Saudi Arabia

**Keywords:** intensive care, pediatric asthma, severe acute asthma, status asthmaticus, Pediatric Intensive Care Unit (PICU), respiratory failure

## Abstract

**Highlights:**

**What are the main findings?**

- Severe pediatric asthma requiring PICU admission predominantly affects young children and is associated with poor pre-admission disease control and low medication adherence.
- Nutritional status, including both undernutrition and overweight/obesity, is associated with severe asthma presentations.

**What are the implications of the main finding?**

- Strengthening outpatient asthma management, including adherence support and caregiver education, may help reduce preventable PICU admissions.
- Integrating routine nutritional assessment into asthma care may improve risk stratification and clinical outcomes.

**Abstract:**

Background: Severe asthma exacerbations remain a major cause of pediatric intensive care unit (PICU) admissions, particularly in early childhood. Objective: To describe the demographic characteristics, clinical features, management strategies, and short-term outcomes of children admitted to the PICU with severe acute asthma exacerbations. Methods: A retrospective descriptive study was conducted of pediatric patients aged 1–14 years with severe acute asthma requiring PICU admission at King Salman Medical City, Madinah, Saudi Arabia (January 2023–October 2024). A total of 73 patients were included. Data included demographics, risk factors, medical history, clinical presentation, management, and outcomes. Results: The mean patient age was 4.6 years, with most (57.5%) aged 1–5 years. Males comprised 56.2% of cases. WHO BMI-for-age z-score assessment revealed a bimodal nutritional distribution: 27.9% of patients were underweight, including 20.6% with severe underweight, while 29.4% were overweight or obese; 42.6% had normal nutritional status. Severe undernutrition was concentrated in the 1–5-year age group, whereas obesity predominated in the 6–10-year age group. A family history of asthma was noted in 54.8% of patients; 16.4% had prior COVID-19 infection. Early symptom onset and delayed diagnosis were common. Poor asthma control was documented in 60.3%, with low medication adherence (9.6%) and limited aerochamber use (13.7%). The most frequent presenting symptoms were dyspnea, cough, and wheezing. Management followed evidence-based protocols: systemic corticosteroids and bronchodilators were first-line therapies. The mean PICU stay was 3.1 days and the mean hospital stay was 8.1 days. No mortality or major complications occurred; 93.2% of patients were discharged in good health. Conclusions: Severe pediatric asthma requiring PICU admission is associated with early symptom onset, a bimodal pattern of nutritional risk encompassing both undernutrition and overweight/obesity, family history of asthma, and inadequate outpatient management. These descriptive findings highlight the need for age-adjusted nutritional screening, enhanced medication adherence support, and targeted outpatient education to reduce avoidable PICU admissions.

## 1. Introduction

Severe acute asthma is a major pediatric emergency that requires prompt recognition and aggressive treatment to prevent serious complications and mortality. Despite significant advances in asthma management, severe exacerbations continue to contribute substantially to pediatric morbidity and healthcare utilization, particularly among younger children who may deteriorate rapidly [1,2]. Pediatric asthma represents a growing global health burden, with prevalence varying across geographic regions. In Saudi Arabia and other Middle Eastern countries, environmental and climatic factors, including desert dust exposure, high temperatures, low humidity, and frequent sandstorms, may contribute to increased asthma prevalence and severe exacerbations among children [3,4,5].

Children admitted to the PICU for severe acute asthma represent a high-risk subgroup characterized by significant respiratory compromise and the need for intensive monitoring and advanced supportive care [6]. Clinical presentation may include marked airway obstruction, increased work of breathing, hypoxemia, and, in severe cases, the need for ventilatory support. Such critically ill children demand special attention and individualized treatment strategies are required to optimize outcomes [7].

Treatment of severe acute asthma in children has also developed significantly, with evidence-based changes in practice highlighting the importance of early aggressive bronchodilator, systemic corticosteroid, and supportive care interventions. Despite standardized treatment protocols, variability in outcomes persists, suggesting that patient characteristics, timing of intervention, and institutional practices may influence prognosis [8,9].

Data describing the characteristics and outcomes of children admitted to the PICU with severe asthma in Saudi Arabia remain limited. The region’s peculiarities, including demographic, environmental, and healthcare system characteristics, necessitate region-specific studies to understand disease patterns and optimal management. Madinah serves a diverse population with varying socioeconomic and environmental exposures, providing an appropriate setting for evaluating severe pediatric asthma requiring intensive care [10].

Identifying clinical patterns, risk factors, and treatment responses may help improve prevention strategies and support evidence-based management of severe pediatric asthma in similar healthcare settings [11,12]. Therefore, this study aimed to characterize the demographic features, clinical presentation, management strategies, and short-term outcomes of children admitted to the PICU with severe acute asthma in Madinah, Saudi Arabia. The findings may help strengthen the regional evidence base on severe pediatric asthma in similar healthcare settings.

## 2. Materials and Methods

### 2.1. Study Design and Setting

This retrospective descriptive study included children admitted to the Pediatric Intensive Care Unit (PICU) at King Salman Medical City in Madinah, Saudi Arabia, with severe acute asthma. The primary objective was to describe the clinical profile, pre-admission management, ICU treatment, and short-term outcomes of this population. Secondary exploratory subgroup comparisons were performed for age and nutritional status where data allowed.

### 2.2. Study Population and Inclusion Criteria

The study population included all children aged 1–14 years admitted to the PICU between January 2023 and October 2024 with a primary diagnosis of severe acute asthma exacerbation. Inclusion criteria comprised children aged 1–14 years admitted with severe acute asthma or status asthmaticus requiring PICU-level care, as determined by the attending pediatric intensivist, and with complete medical records available for review. Severe acute asthma was defined according to GINA criteria: marked breathlessness (inability to complete sentences), use of accessory respiratory muscles, oxygen saturation below 92% on room air, or peak expiratory flow/FEV1 below 40% predicted. PICU admission was indicated by failure to respond to initial emergency treatment (inhaled bronchodilators and systemic corticosteroids), requirement for continuous bronchodilator nebulization, or clinical signs of impending respiratory failure.

### 2.3. Definitions

Asthma control: Control status before PICU admission was classified using documentation from the medical record and the treating team’s assessment. Good control referred to the absence of frequent daytime or nighttime symptoms, no recent exacerbations requiring urgent care, and no limitation of usual activity. Poor/uncontrolled asthma is referred to recurrent symptoms, recent exacerbations, or documented clinician assessment of uncontrolled disease. Medication adherence: Good adherence was defined as documented regular use of prescribed controller and/or reliever therapy as instructed, without missed doses noted in the chart. Poor adherence was defined as irregular use, omission of prescribed therapy, or explicit noncompliance documented in the record. Risk factor awareness: Awareness of asthma-related risk factors was defined as documented caregiver or patient recognition of common triggers such as viral infection, tobacco smoke, dust, air pollution, exercise, or cold air. Nutritional status: Weight status was categorized using BMI-for-age z-scores according to WHO standards. Delayed diagnosis: A gap of more than six months between the documented age of first asthma-like symptom onset (as recorded in the medical history) and the age at formal asthma diagnosis was classified as delayed diagnosis. Age at symptom onset and age at diagnosis were both extracted from the medical record. Poor asthma control (as a discrete variable): In addition to the clinician-assigned control classification described above, poor control was operationally identified by the presence of any of the following in the six months before admission: two or more unscheduled healthcare visits, one or more oral steroid courses, or documented limitation of daily activity attributable to asthma symptoms.

### 2.4. Exclusion Criteria

Patients were excluded if they had concurrent pneumonia or other significant respiratory infections, chronic lung disease unrelated to asthma, congenital heart disease with significant left-to-right shunts, immunodeficiency disorders, incomplete medical records, or transfer to or from another ICU. Children presenting with isolated viral-induced wheeze or first-time wheezing episodes without clinical features suggestive of asthma were excluded from the study, to ensure the cohort represented established asthma exacerbations rather than alternative wheezing phenotypes.

### 2.5. Data Collection

Trained research personnel systematically reviewed the medical records of eligible patients using a standardized data collection form. Extracted variables included demographic characteristics, clinical presentation, disease severity, laboratory findings, imaging results, treatment interventions, complications, and clinical outcomes.

### 2.6. Statistical Analysis

Statistical analyses were performed using SPSS version 16.0 (SPSS Inc., Chicago, IL, USA). Descriptive statistics were primarily used to summarize patient characteristics, clinical presentation, management, and outcomes. Exploratory subgroup comparisons were performed selectively where clinically relevant. Continuous variables were summarized as means ± standard deviations for normally distributed data or medians with interquartile ranges for non-normally distributed data. Categorical variables were presented as frequencies and percentages. Exploratory subgroup analyses were performed to evaluate associations between WHO BMI-for-age categories and selected demographic, clinical, laboratory, and outcome variables. Continuous variables were compared using the Kruskal–Wallis test, while categorical variables were analyzed using the Chi-square test or Fisher’s exact test when appropriate. A *p*-value < 0.05 was considered statistically significant. Given the retrospective, descriptive nature of this study and the relatively small sample size, these comparisons should be interpreted with caution. No adjustments for multiple comparisons were applied.

## 3. Results

A total of 73 pediatric patients admitted to the PICU with severe asthma exacerbations were included in the study. The mean age was 4.58 ± 2.90 years, with most patients aged 1–5 years (57.5%), followed by those aged 6–10 years (39.7%). There was a slight male predominance, with males accounting for 56.2% of cases (Table 1). Anthropometric measurements revealed a mean weight of 16.66 ± 8.56 kg, with most patients (60.3%) weighing 10–20 kg. Slightly more than half of the patients had a height < 100 cm. The mean BMI-for-age z-score was −0.49 ± 3.14 (median −0.47), reflecting a population that spans the full spectrum of nutritional extremes. The complete distribution is presented in Table 1. When combining categories, 19 patients (27.9%) were classified as underweight (z < −2), including 14 (20.6%) with severe underweight (z < −3), consistent with acute or chronic malnutrition. Conversely, 20 patients (29.4%) were overweight or obese (z > +1), with 14 (20.6%) meeting the WHO threshold for obesity (z > +2). Only 29 patients (42.6%) fell within the normal nutritional range (z −2 to +1). Subgroup analysis by age revealed that undernutrition was predominantly concentrated in the 1–5-year age group, which had a mean z-score of −0.82 ± 3.64 and accounted for 12 of the 14 severely underweight patients. In contrast, the 6–10-year group showed a higher mean z-score of +0.81 ± 3.93, with obesity more prevalent (9 of 16 cases classified as overweight or obese). Both male and female patients were similarly affected, with comparable proportions of undernutrition and obesity across sexes. Exploratory subgroup analysis demonstrated significant differences across WHO BMI-for-age categories for age at asthma diagnosis (Kruskal–Wallis test, *p* = 0.037) and base excess values on blood gas analysis (*p* = 0.048). Underweight children tended to have an earlier age of asthma diagnosis compared with overweight or obese patients. Variations in base excess values were also observed between BMI groups, suggesting possible differences in metabolic compensation during severe asthma exacerbations. No statistically significant associations were identified between BMI category and PICU length of stay, oxygen support requirements, previous ICU admissions, or most other clinical variables. Most patients had no comorbidities (63%), while 16.4% had multiple comorbidities. A family history of asthma was present in 54.8% of patients, most commonly among siblings (Table 1). A family history of other allergic conditions was absent in 41.1% of patients, whereas 11% reported a family history of eczema. Prior COVID-19 infection was documented in 16.4% of patients before ICU admission (Table 1).

Asthma symptoms began at a mean age of 1.9 ± 1.6 years, while diagnosis occurred at a mean age of 2.37 ± 1.68 years. Most patients (80.8%) had a confirmed asthma diagnosis before PICU admission, whereas 19.2% were diagnosed during hospitalization. Prior healthcare utilization was limited, with only 37% of patients having previous hospital visits, most commonly to Primary Healthcare Centers (PHCs) and General Pediatric Centers. Medication adherence was poor, with only 9.6% of patients demonstrating good compliance with prescribed therapy. Regular treatment before ICU admission was documented in 31.5% of patients, primarily involving salbutamol and combination inhalers containing corticosteroids. Spacer device (aerochamber) use was low (13.7%). Previous emergency department visits for asthma exacerbations were reported in 9.6% of patients. Hospital admissions due to asthma exacerbation occurred in 41.1% of patients, with a mean of 2.18 ± 1.65 admissions and an average length of stay of 5.25 ± 2.01 days. Prior ICU admission was reported in 20.5% of patients, while 16.4% had previously required supplemental oxygen therapy, most commonly delivered via nasal cannula or non-rebreather mask, with some patients receiving high-flow nasal cannula support. Asthma control prior to admission was generally poor, with 60.3% of patients classified as uncontrolled and only 2.7% achieving good control. Prior systemic steroid use was documented in 37% of patients, with a mean of 2.11 ± 1.39 steroid courses and an average treatment duration of 3.62 ± 0.88 days per course (Table S1).

The most common presenting symptoms were dyspnea (98.6%), cough (95.9%), and fever (60.3%). Less frequent symptoms included decreased level of consciousness, vomiting, and rhinorrhea. Decreased air entry was identified in 87.7% of patients during physical examination. Wheezing was present in 87.7% of patients. Chest radiography was performed in 89% of patients, whereas chest computed tomography was used less frequently (4.1%). Serum IgE levels were assessed in 6.8% of patients. Additional investigations included septic screening, blood cultures, and MRSA surveillance when clinically indicated, as summarized in Table S2 in Supplementary Materials. Physiological assessment demonstrated tachycardia, with a mean heart rate of 140.75 ± 19.74 beats/min. Mean systolic and diastolic blood pressures were 102.32 ± 10.69 mmHg and 61.21 ± 11.43 mmHg, respectively. The mean body temperature was 37.39 ± 0.74 °C. Mean oxygen saturation was reduced on room air (85.77 ± 6.68%) and improved following oxygen supplementation and supportive interventions.

Hematological analysis is shown in Table S3 in the Supplementary Materials. Blood gas analysis demonstrated mild acid–base disturbance, with a mean pH of 7.31 ± 0.13, bicarbonate level of 20.74 ± 4.33 mmol/L, PCO_2_ of 39.43 ± 7.44 mmHg, and base excess of −5.38 ± 7.50 mmol/L. Mean partial pressure of oxygen was 60 ± 24.19 mmHg. Elevated IgE levels were documented in patients tested, with a mean value of 567.35 ± 388.90 IU/L.

Asthma-related functional impact and associated risk factors are illustrated in Table S4 in the Supplementary Materials). Impairment in daily activities was reported in 9.6% of patients, while 2.7% experienced effects on school performance. Awareness of asthma-related risk factors was present in 56.2% of patients. Upper respiratory tract infections were the most commonly identified trigger, followed by environmental exposures including tobacco smoke, air pollution, exercise, and cold air.

The most commonly administered treatment regimen included continuous salbutamol nebulization, ipratropium bromide, systemic corticosteroids, and magnesium sulfate (42.5%). Systemic corticosteroids were widely used, with intravenous methylprednisolone followed by oral prednisolone therapy. The mean duration of systemic steroid therapy was 5.52 ± 2.50 days, with minimal adverse effects reported (1.4%). Oxygen therapy was required in all patients. Multiple oxygen delivery approaches were employed, with a mean treatment duration of 5.05 ± 3.70 days. All patients required oxygen supplementation during PICU admission. Conventional oxygen therapy was most commonly delivered using non-rebreather oxygen masks in 35 patients (47.9%), followed by face masks in 22 patients (30.1%) and nasal cannula support in 14 patients (19.2%). Escalation to advanced respiratory support was required in a subset of patients, including high-flow nasal cannula (HFNC) in 11 patients (15.1%), noninvasive mechanical ventilation in 7 patients (9.6%), invasive mechanical ventilation in 5 patients (6.8%), and high-frequency oscillatory ventilation (HFOV) in 8 patients (11.0%). Because some patients required escalation between multiple respiratory support modalities during their PICU course, percentages exceeded 100%. Although culture-confirmed infection was uncommon, empirical antibiotic therapy was administered (Table S5).

Common radiographic findings included hyperinflation and bilateral infiltrative changes. Chest CT imaging identified congenital anomalies in a small number of patients, as described in Table S5. Laboratory cultures were negative in most cases, with only 2.7% of patients demonstrating positive culture results. No major complications or mortality were observed during the study period. The mean PICU stay was 3.15 ± 2.16 days, while total hospital stay averaged 8.13 ± 5.70 days. Most patients (95.9%) were discharged, of whom 93.2% were reported to be in good health, whereas 4.1% required referral for specialized follow-up care (Table S5).

## 4. Discussion

The mean age of patients was 4.58 years, with a majority (57.5%) aged between 1 and 5 years, indicating that early childhood is a critical period for severe asthma manifestations requiring ICU admission. This is consistent with previous studies that suggest children under five are particularly vulnerable to asthma exacerbations due to immature airway development and high susceptibility to viral infections [13]. A male predominance (56.2%) was noted, although not statistically significant, aligning with the literature that asthma prevalence is higher in boys during early childhood [14].

Nutritional status emerged as an essential factor; the WHO BMI-for-age z-score assessment demonstrated a bimodal nutritional distribution within this pediatric asthma PICU cohort, with 27.9% classified as underweight and 29.4% as overweight or obese. This finding highlights the coexistence of undernutrition and obesity in children with severe asthma and reflects the broader nutritional transition reported in middle-income settings. Undernutrition was concentrated among younger children and may be associated with increased vulnerability to severe asthma exacerbations because of impaired immune function, reduced respiratory muscle strength, and delayed lung development, although causality cannot be established from this observational study. Conversely, obesity represented a clinically important subgroup that may be associated with increased asthma severity through mechanical restriction, systemic inflammation, and reduced treatment responsiveness. Previous studies suggest that both undernutrition and obesity may adversely affect respiratory function and asthma outcomes, supporting the importance of nutritional assessment in asthma management [15].

Although most children had no comorbidities, allergic conditions such as eczema were present in a subset of patients, consistent with the recognized association between atopy and asthma [16]. More than half of the cohort reported a family history of asthma, supporting the role of genetic susceptibility in asthma pathogenesis [17]. Prior COVID-19 infection was also documented in some patients, although its relationship with pediatric asthma severity remains uncertain [18,19,20].

Beyond baseline demographic characteristics, healthcare utilization patterns provided additional insight into disease management prior to ICU admission. The early onset of symptoms (mean age 1.9 years) and subsequent diagnosis (2.37 years) suggest a pattern of early asthma development. While most children (80.8%) had a pre-existing asthma diagnosis, nearly one-fifth were diagnosed during their ICU stay—highlighting gaps in early detection. Prior studies describe a high burden of pediatric status asthmaticus in ICUs and emphasize the challenge of reaching a definitive diagnosis in emergency scenarios [21]. Most patients had limited pre-ICU healthcare utilization and were managed primarily in primary healthcare or general pediatric settings rather than specialized asthma clinics. Medication adherence, maintenance therapy use, and aerochamber utilization were notably low, suggesting important gaps in long-term outpatient asthma management and caregiver education, consistent with previous reports of inadequate outpatient care among children requiring ICU admission for severe asthma [22].

Control of asthma before ICU admission was overwhelmingly poor—60.3% were uncontrolled, and only 2.7% were well-controlled. Such findings reaffirm that poor asthma control is a major predictor of critical exacerbations and ICU admissions and further highlight deficiencies in early intervention and education, similar to patterns described by GINA (2024) regarding inadequate asthma education and follow-up associated with increased hospitalizations [23].

A substantial proportion of patients (41.1%) had previous asthma-related hospitalizations, ICU admissions, or prior respiratory support, suggesting a high disease burden and recurrent severe exacerbations requiring closer outpatient follow-up [24]. Prior systemic steroid use before ICU transfer was relatively limited, which may reflect delayed recognition or undertreatment of worsening asthma symptoms [25].

The clinical presentation was dominated by respiratory symptoms, particularly dyspnea, cough, and wheezing, consistent with the typical presentation of severe pediatric asthma exacerbations. Fever was also common, as it is a well-documented trigger for severe exacerbations in children.

Chest radiography was frequently performed to exclude complications, while blood gas abnormalities and reduced oxygen saturation on room air reflected significant respiratory compromise consistent with severe pediatric asthma exacerbations. Improvement after oxygen supplementation highlights the importance of early respiratory support in critically ill patients [8,23].

Management strategies followed evidence-based protocols for severe asthma, utilizing systemic corticosteroids, inhaled bronchodilators, and adjunct therapies such as magnesium sulfate. Methylprednisolone was the preferred systemic steroid (57.7%), consistent with recommendations for moderate to severe exacerbations. The use of multiple oxygen delivery methods, particularly non-rebreather masks (34.2%), reflects the severity of hypoxia in these patients. The incidence of side effects was minimal (1.4%). Respiratory support requirements reflected the severity of respiratory compromise among children admitted to the PICU with severe asthma exacerbations. All patients required supplemental oxygen therapy, most commonly via non-rebreather oxygen masks and face masks. Advanced respiratory support, including high-flow nasal cannula (HFNC), noninvasive ventilation, invasive mechanical ventilation, and high-frequency oscillatory ventilation (HFOV), was required in a subset of patients. The relatively low proportion requiring invasive ventilation may indicate the effectiveness of early aggressive management and supportive care in preventing progression to severe respiratory failure. Nevertheless, the need for advanced ventilatory support in some patients highlights the potential for rapid clinical deterioration in severe pediatric asthma.

Although microbiologically confirmed infection was uncommon, empirical antibiotic therapy remained frequent [26]. PICU and hospital lengths of stay were comparable to previous reports of severe pediatric asthma admissions [27,28,29]. Most patients were discharged in good health, although the low reported impact on daily activities and school performance may reflect limited follow-up or reporting bias. The absence of mortality and major complications in this cohort is reassuring and may reflect timely PICU intervention and standardized management approaches. However, the relatively short follow-up period and retrospective study design limit conclusions regarding long-term respiratory outcomes, recurrence risk, and subsequent healthcare utilization.

This study provides one of the few region-specific descriptions of pediatric severe asthma requiring ICU admission in Saudi Arabia. The inclusion of clinical presentation, physiologic parameters, treatment approaches, and short-term outcomes offers a comprehensive characterization of critically ill pediatric asthma patients in this setting.

## 5. Conclusions

In this cohort of pediatric patients with ICU admissions for asthma, we identified a concerning convergence of preventable risk factors, including medication non-adherence, the absence of controller therapy, and nutritional status. Addressing these potentially modifiable factors may help improve health outcomes and reduce future admissions. The absence of mortality despite disease severity suggests that earlier intervention targeting modifiable factors may reduce the likelihood of ICU admission. These findings highlight the need for targeted interventions to improve outpatient asthma management and reduce preventable severe exacerbations.

### Study Limitations

Potential limitations of this study design were recognized, including the retrospective nature of data collection, potential incomplete documentation in medical records, a single-center design that limited generalizability, and the lack of long-term follow-up data. In the interpretation and discussion of the study results, these limitations were taken into consideration.

## Figures and Tables

**Table 1:** Demographic and Baseline Characteristics of Pediatric Patients Admitted to the PICU with Severe Asthma (N = 73).

Variable	n	%/Mean ± SD
Age and Sex		
Mean age (years)	—	4.58 ± 2.90
Age 1–5 years	42	57.5
Age 6–10 years	29	39.7
Age >10 years	2	2.7
Male sex	41	56.2
Female sex	32	43.8
Anthropometric Characteristics		
Mean weight (kg)	—	16.66 ± 8.56
Mean height (cm)	—	99.40 ± 19.98
Mean BMI (kg/m^2^)	—	17.52 ± 8.32
WHO BMI-for-Age Z-Score Classification (n = 68) *		
Mean BMI-for-age z-score	—	−0.49 ± 3.14
Severely Underweight (Z < −3)	14	20.6
Underweight (Z −3 to −2)	5	7.4
Normal weight (Z −2 to +1)	29	42.6
Overweight (Z +1 to +2)	6	8.8
Obese (Z > +2)	14	20.6
Combined underweight (Z < −2)	19	27.9
Combined overweight/obese (Z > +1)	20	29.4
Comorbidities		
No comorbidities	46	63.0
Multiple comorbidities	12	16.4
Laryngomalacia	2	2.7
Allergic rhinitis	2	2.7
Congenital heart disease	1	1.4
Rickets	1	1.4
Food allergy	1	1.4
Insulin-dependent diabetes mellitus	1	1.4
Mitral valve prolapse with tachyarrhythmia	1	1.4
Hydrocephalus	1	1.4
Mild-to-moderate neutropenia	1	1.4
Atopic dermatitis (eczema)	1	1.4
Allergic bronchopulmonary aspergillosis	1	1.4
Congenital diaphragmatic hernia	1	1.4
Family History of Asthma		
Family history of asthma	40	54.8
No family history of asthma	17	23.3
Siblings	11	15.1
Mother	5	6.8
Father	4	5.5
Grandmother	3	4.1
Other relatives	3	4.1
Grandfather	1	1.4
Multiple relatives	9	12.3
Family History of Allergic Disease		
Eczema	8	11.0
Food allergy	3	4.1
Allergic rhinitis	3	4.1
Multiple allergic conditions	3	4.1
No family history of allergy	30	41.1
Previous COVID-19 Infection		
Prior documented COVID-19 infection	12	16.4
No prior COVID-19 diagnosis	2	2.7

Abbreviations: PICU = Pediatric Intensive Care Unit; BMI = Body Mass Index; SD = Standard Deviation; COVID-19 = Coronavirus Disease 2019. * WHO BMI-for-age z-scores calculated using LMS method. Percentages are based on available data and may not total 100% due to incomplete documentation.

## Data Availability

The raw data supporting the conclusions of this article will be made available by the authors on request.

## Supplementary Materials

The following supporting information can be downloaded at https://www.mdpi.com/article/10.3390/children13050710/s1: Table S1: Previous Clinical History, Diagnostic Status, and Asthma Management Characteristics Among Children Admitted to the PICU; Table S2: Summary of clinical presentation, diagnosis modalities, and severity of asthma; Table S3: Summary of vital signs, hematological parameters, and blood gas analysis in pediatric asthma patients admitted to the Intensive Care Unit; Table S4: Impact of asthma on the patient’s performance and identified risk factors that trigger asthma; Table S5: Summary of management strategies, including pharmacological interventions, mechanical ventilation, and adjunctive therapies, and outcomes for ICU length of stay.
