# Supplementary material for: Severe Asthma Exacerbations in the Pediatric Intensive Care Unit: Clinical Profile, Management, and Outcomes—Retrospective Study
Source: Children (Basel). 2026 May 21;13(5):710. doi: 10.3390/children13050710 (PMC13205015; doi:10.3390/children13050710)
Supplement: Supplementary file 1 [file children-13-00710-s001.zip › children-4326726-supplementary.pdf]

Table S1. Previous Clinical History, Diagnostic Status, and Asthma Management Characteristics Among Children Admitted to the PICU.

Variable	n	% / Mean ± SD
Age at first symptoms (years)	—	1.9 ± 1.6
Age at diagnosis (years)	—	2.37 ± 1.68
Diagnosis confirmed before ICU admission	59	80.8
Diagnosis made during ICU admission	14	19.2
Healthcare Utilization		
Previous hospital visits	27	37.0
No previous hospital visits	46	63.0
Primary Healthcare Center (PHC)	14	19.2
General pediatric clinic	10	13.7
Allergist clinic	1	1.4
Pulmonology clinic	1	1.4
Multiple healthcare facilities	2	2.7
Previous emergency department visits	7	9.6
Mean number of emergency visits	—	3.42 ± 2.06
Asthma Management Before ICU Admission		
Regular medication use before ICU admission	23	31.5
No regular medication use	45	61.6
Salbutamol and inhaled corticosteroid combination	15	20.5
Salbutamol only	8	11.0
Spacer (aerochamber) use	10	13.7
Medication adherence (good compliance)	7	9.6
Previous Asthma Severity and Healthcare Burden		
Previous hospital admission for asthma exacerbation	30	41.1
Number of hospital admissions	—	2.18 ± 1.65
Mean hospital length of stay (days)	—	5.25 ± 2.01
Previous ICU admission	15	20.5
Previous supplemental oxygen or respiratory support	12	16.4
Nasal cannula / non-rebreather mask	12	16.4
High-flow nasal cannula (HFNC)	7	9.6
Asthma Control Before Admission		
Well-controlled asthma	2	2.7

Partially controlled asthma	8	11.0
Uncontrolled asthma	44	60.3
Prior Steroid Exposure		
Previous systemic steroid use	27	37.0
Number of steroid courses	—	2.11 ± 1.39
Duration of steroid courses (days)	—	3.62 ± 0.88

Abbreviations: PICU = Pediatric Intensive Care Unit; ICU = Intensive Care Unit; PHC = Primary Healthcare Center; HFNC = High-Flow Nasal Cannula; SD = Standard Deviation. Note: Percentages may not total 100% because of missing or incomplete documentation

Table S2. Summary of clinical presentation, diagnosis modalities, and severity of asthma.

Variables	Frequency	% age
**Symptom**		
Cough	70	95.9
Fever	44	60.3
Dyspnea	72	98.6
Decrease LOC	6	8.2
Vomiting	6	8.2
Runny nose	10	13.7
**Clinical signs**		
Decreased **Air entry**	64	87.7
**Distress level**		
Moderate	29	39.7
Severe	34	46.6
**Chest sound**		
Wheezing	64	87.7
**Imaging modalities**		
**Chest X-ray**		
Yes	65	89
No	8	11
**Chest CT**		
Yes	3	4.1
No	70	98.9
**IgE performed**		
Yes	5	6.8
**Lab tests**		
Septic workup	14	19.2
Peripheral blood culture+axillary/groin/nasal swabs for MRSA	8	11
Peripheral blood culture	4	5.5
Lactate	3	4.1
Positive respiratory syncytial virus/Staphylococcus/coagulase	1	1.4
MRSA culture	1	1.4
Urine C/S	1	1.4

Abbreviations: CT: Computed Tomography, MRSA: Methicillin Resistant *Staphylococcus aureus*, C/S: Culture/Sensitivity, LOC: Level of Consciousness

Table S3. Summary of vital signs, hematological parameters, and blood gas analysis in pediatric asthma patients admitted to the Intensive Care Unit.

Variables	N	Mean	SD
Heart rate (beats /minute)	73	140.75	19.74
Systolic BP (mmHg)	73	102.32	10.69
Diastolic BP (mmHg)	73	61.21	11.43
Temperature (^{0}C)	73	37.39	0.74
Oxygen saturation in room air (%)	71	85.77	6.68
Oxygen saturation oxygen (%)	72	96.76	3.13
Oxygen saturation after life support (%)	73	95.95	11.42
CBCD	73	14.67	5.91
Base excess (mmol/L)	41	-5.38	7.50
pH	73	7.31	0.13
Neutrophil (cells/mcL)	73	11.21	5.58
Lymphocytes (cells/µL)	73	2.51	2.29
Eosinophils (cells/ µL)	73	0.10	0.20
Hemoglobin (g/dL)	73	11.67	1.45
Platelets (mm^3)	73	386.27	155.06
U&E (mmol/L)	73	139.38	4.04
K (mmol/L)	73	4.49	0.70
Mg (mmol/L)	54	1.04	0.22
Blood gas	73	0.00	0.00
PCO_2 (mmHg)	73	39.43	7.44
O_2	10	60	24.19
HCO_3 (mmol/L)	73	20.74	4.33
IgE (IU/mL)	4	567.35	388.90

Abbreviations: K: Potassium, Mg: Magnesium, O_2: Oxygen, HCO_3: Bicarbonate, PCO_2: Partial Pressure of carbon dioxide, U&E: Urea & Electrolyte, BP: Blood Pressure

Table S4. Impact of asthma on the patient's performance and identified risk factors that trigger asthma.

Variables	Frequency	% age
**Asthma impaired daily activities**		
Yes	7	9.6
No	18	34.7
**Asthma affected school performance**		
Yes	2	2.7
No	16	21.9
**Do you know triggers for asthma**		
Yes	41	56.2
No	27	37
**If yes, what are the known triggers**		
URTI	23	31.5

Allergy	2	2.7
Strong odors	1	1.4
Multiple (smoking, air pollution, exercise, cold air)	16	21.9

Abbreviation: URTI: Upper respiratory Tract Infection

Table S5. Summary of management strategies, including pharmacological interventions, mechanical ventilation, and adjunctive therapies, and outcomes for ICU length of stay.

Variables	Frequency	% age
**Medication used for the management of asthma**		
Continuous salbutamol inhaler, Ipratropium bromide inhaler, Systemic steroid, Magnesium sulfate	31	42.5
Continuous salbutamol inhaler, Systemic steroid, Magnesium sulfate	18	24.7
Others	24	32.9
**Systemic steroids are used for the management of asthma**		
Methylprednisolone	42	57.5
Methylprednisolone IV + Oral prednisolone	28	38.4
Hydrocortisone IV	1	1.4
Methylprednisolone IV + Dexamethasone	1	1.4
Methylprednisolone IV + Oral Prednisolone and Dexamethasone	1	1.4
**Duration of systemic steroids (Days) (Mean±SD)**	5.52±2.50	
**Side effects of systemic steroids**		
Yes	1	1.4
No	25	34.2
**Need for oxygen support***		
Non-rebreather oxygen mask	35	47.9
Face mask	22	30.1
Nasal cannula	14	19.2
High-flow nasal cannula (HFNC)	11	15.1
High-frequency oscillatory ventilation (HFOV)	8	11.0
**Duration of oxygen therapy (Days) (Mean±SD)**	5.05±3.70	
**Culture proven sepsis**		
Yes	1	1.4
No	72	98.6
**Antibiotics used for the management of asthma**		
Yes	73	100
**Chest X-ray outcomes**		
Hyperinflation	16	21.9
Hyperinflation with bilateral infiltration	15	20.5
Bilateral infiltration	8	11
Hyperinflation with mild diffuse infiltration	7	9.6
Hyperinflation with right side infiltration	1	1.4
Hyperinflation + Bilateral infiltration more on the right side	1	1.4
Bilateral diffuse infiltrations	1	1.4
Others	16	21.9
**Chest CT outcomes**		

Anterior diaphragmatic (Morgagni) hernia associated with patchy air space disease	1	1.4
Innominate artery compression syndrome	1	1.4
Picture of pulmonary arterial hypertension, bilateral small lung volumes with pulmonary changes for clinical correlation	1	1.4
**Lab (culture) outcomes**		
Negative	20	27.4
Positive	2	2.7
**Complications**		
No	73	100
**Mortality**		
No	73	100
**PICU LOS (Mean±SD)**	3.15±2.16	
**Hospital LOS (Mean±SD)**	8.13±5.70	
**Status**		
Discharged	70	95.9
Referred (cardiology, DAMA)	3	4.1
**If discharged**		
Healthy	68	93.2
Not healthy	3	4.1

Abbreviations: SD: Standard Deviation, HFOV: High Frequency Oscillatory Ventilation, HFNC: High Flow Nasal Cannula ICU: Intensive Care Unit, SD: Standard Deviation, CT: Computed Tomography, DAMA: Discharge Against Medical Advice, LOS: Length of Stay

*Percentages are based on the total study population (n = 73). Patients may have received more than one respiratory support modality during PICU admission; therefore, percentages exceed 100%.
